# TILLING by sequencing to identify induced mutations in stress resistance genes of peanut (*Arachis hypogaea*)

**DOI:** 10.1186/s12864-015-1348-0

**Published:** 2015-03-07

**Authors:** Yufang Guo, Brian Abernathy, Yajuan Zeng, Peggy Ozias-Akins

**Affiliations:** Department of Horticulture, University of Georgia -Tifton Campus, 2360 Rainwater Rd, Tifton, GA 31793-5766 USA; Center for Applied Genetic Technologies, University of Georgia, 111 Riverbend Road, Athens, GA 30602 USA

## Abstract

**Background:**

Targeting Induced Local Lesions in Genomes (TILLING) is a powerful reverse genetics approach for functional genomics studies. We used high-throughput sequencing, combined with a two-dimensional pooling strategy, with either minimum read percentage with non-reference nucleotide or minimum variance multiplier as mutation prediction parameters, to detect genes related to abiotic and biotic stress resistances. In peanut, lipoxygenase genes were reported to be highly induced in mature seeds infected with *Aspergillus spp*., indicating their importance in plant-fungus interactions. Recent studies showed that phospholipase D (PLD) expression was elevated more quickly in drought sensitive lines than in drought tolerant lines of peanut. A newly discovered lipoxygenase (LOX) gene in peanut, along with two peanut PLD genes from previous publications were selected for TILLING. Additionally, two major allergen genes *Ara h 1* and *Ara h 2*, and fatty acid desaturase *AhFAD2*, a gene which controls the ratio of oleic to linoleic acid in the seed, were also used in our study. The objectives of this research were to develop a suitable TILLING by sequencing method for this allotetraploid, and use this method to identify mutations induced in stress related genes.

**Results:**

We screened a peanut root cDNA library and identified three candidate LOX genes. The gene *AhLOX7* was selected for TILLING due to its high expression in seeds and roots. By screening 768 M2 lines from the TILLING population, four missense mutations were identified for *AhLOX7*, three missense mutations were identified for *AhPLD*, one missense and two silent mutations were identified for *Ara h 1.01*, three silent and five missense mutations were identified for *Ara h 1.02*, one missense mutation was identified for *AhFAD2B*, and one silent mutation was identified for *Ara h 2.02*. The overall mutation frequency was 1 SNP/1,066 kb. The SNP detection frequency for single copy genes was 1 SNP/344 kb and 1 SNP/3,028 kb for multiple copy genes.

**Conclusions:**

Our TILLING by sequencing approach is efficient to identify mutations in single and multi-copy genes. The mutations identified in our study can be used to further study gene function and have potential usefulness in breeding programs.

**Electronic supplementary material:**

The online version of this article (doi:10.1186/s12864-015-1348-0) contains supplementary material, which is available to authorized users.

## Background

Peanut (*Arachis hypogaea* L.) is one of the world’s most important oil seed crops. However, more than 70% of the peanut growing area is in arid and semi-arid regions. Abiotic stress caused by drought, as well as the co-occurring biotic stress due to *Aspergillus flavus* invasion of pods reduces plant productivity and the quality of seeds used for human consumption.

Using traditional breeding methods, several cultivars were developed for abiotic and biotic stress resistances, such as for nematode resistance [1] and tomato spotted wilt virus (TSWV) resistance [2-5]. However, due to the scarcity of drought and salt tolerance alleles in the peanut gene pool, the quantitative nature of abiotic stress tolerance, and the difficulty of selection, peanut stress resistance breeding can be a time consuming, labor-intensive, and difficult process [6].

Targeting Induced Local Lesions in Genomes (TILLING) is a powerful reverse genetics approach for functional genomics studies. It has been widely used to study gene function in various organisms including *Brassica napus* [7,8], *Brassica rapa* [9], *Lotus japonicus* [10], *Zea mays* [11], *Oryza sativa* [12], and *Drosophila* [13]. Traditional TILLING involves PCR amplification followed by digestion with CEL1 nuclease, a mismatch-specific nuclease that can recognize and cut heteroduplex DNA to identify single nucleotide substitutions or small insertions/deletions [14]. Alternatively, direct sequencing, high-performance liquid chromatography (HPLC), regular electrophoresis, high-resolution melting (HRM), and MALDI-TOF can also be used for TILLING applications [15]. Next-generation sequencing (NGS) has been reported as the latest mutation detection method to screen TILLING populations [16,17]. Previous reports using Illumina sequencing for high throughput TILLING not only confirmed known mutations, but also identified several mutants missed from previous heteroduplex detection assays [17]. In this study, we detected mutations in genes related to abiotic and biotic stress resistances in *Arachis hypogaea* using a high-throughput sequencing approach combined with two-dimensional pooling, and updated the TILLING bioinformatics pipeline by implementing either read percentage with non-reference nucleotide (single copy genes) or minimum variance multiplier to set mutation prediction parameters (multi-copy genes).

Lipoxygenases (LOX; EC1.13.11.12) comprise a family of structurally related non-heme iron-containing dioxygenases widely distributed among plants, animals, fungi and bacteria; they catalyse the addition of molecular oxygen to polyunsaturated fatty acids with a cis, cis-1,4-pentadiene structural unit. According to the dioxygenation position of the substrates, plant LOX enzymes can be classified into groups oxygenating polyunsaturated fatty acids at either C-13 (13-LOX) or C-9 (9-LOX) [18]. As a multi-gene family, LOX isoforms have an overall sequence identity of 25-40%. LOX proteins have conserved domains for catalytic iron binding. The catalytic iron is ligated in an octahedral arrangement by three conserved histidines, one *His*/*Asn*/*Ser*, and the C-terminal isoleucine. The LOX family has diversified functions in plants during vegetative growth and development, and contributes to formation of flavor and aroma compounds. In seeds, LOXs can function as storage proteins, while some can also play a role in plant-fungus interactions. In peanut, five lipoxygenase genes have been reported. The gene coding for *AhLOX1* (PnLOX1) [19] is specifically expressed in immature cotyledons, and can be highly induced in mature seeds infected with *Aspergillus spp*., or by methyl jasmonate and wounding. Two other genes in peanut, *AhLOX2*-*3* [20], are also seed specifically expressed, with the highest expression level in mature embryo and immature cotyledons. Although the expression of *AhLOX2*-*3* was increased by wounding, in contrast to *AhLOX1*, both *AhLOX2* and *AhLOX3* were repressed upon *Aspergillus* infection of mature seed. Recently, *LOX4* and *LOX5* genes were described and suggested to also be involved in the response to *A. parasiticus* infection in peanut [21].

Phospholipase D (PLD, EC 3.1.4.4.) is a widely distributed ubiquitous eukaryotic enzyme participating in various cellular processes [22]. It hydrolyses membrane phospholipids to PA (phosphatidic acid) and a free head group such as choline in plants. PA is thought to be an effector in plant physiological processes such as secretion [23], DNA synthesis, and can be converted to second messengers such as diacyglycerol [24]. The active site of PLD consists of four conservative amino acid sequences. Motifs II and IV contain HxKx4Dx6G (G/S) (HDK) and are conserved in all organisms. A minority of PLD family members contain a single HKD motif, while most enzymes of this class contain two HDK motifs for bacterial, plant, yeast, and mammalian sources. Plant PLD normally contains a Ca^2+^ - dependent phospholipid - binding C2 domain and requires Ca^2+^ for activity (C2-PLD) [25], whereas the others may resemble mammalian PLD1 and PLD2, containing adjacent PX (phox homolog) and PH (pleckstrin homology) domains in the N-terminal region of the protein (PX/PH-PLD).

Recent studies have shown that PLD plays an important role in drought and stress tolerance [26-32]. It also plays a role in membrane degradation, seed germination, and acts in signal transduction cascades [25]. In peanut, two types of full-length PLD cDNA, *Ahpld1* and *Ahpld2* have been identified [33,34]. Up-regulation was observed in drought sensitive lines compared with drought tolerant lines of peanut. Southern blot analysis also indicated that PLD is a multigene family in cultivated peanut [33].

In this research, we targeted the above listed peanut stress related genes for cloning or amplification, set up an updated TILLING by sequencing pipeline, and used this pipeline to screen for mutations in an EMS induced population.

## Results and discussion

### Cloning of *Ah LOX* genes expressed in peanut roots

After screening a total of 1.75 × 10^5^ colonies from a root cDNA library, 162 hybridization signals were detected, including 15 high-, 39 medium-, and 108 low- strength. A total of 80 signals including all high- and medium-, and 26 low-strength hybridization signals were selected for secondary screening. Colonies under a single signal were pooled and re-grown on a separate plate. Overall, 80 plates were grown for secondary screening and 61 were identified with hybridization signals, with 14 plates from the “high signal” group, 29 from the “medium signal” group, and 18 from the “low signal” group. A single colony was then picked under each hybridization signal from the secondary screening. Altogether, a total of 135 colonies were picked. Of those, 68 colonies contained recombinant plasmids with insert sizes > 2 kb; 32 colonies showed insert sizes of 1–2 kb. Overall, 77 clones (all 68 with insert sizes >2 kb, and the remaining with inserts between 1 kb and 2 kb) were picked for Sanger sequencing. Ten clones, ranging in size from 1,811 to 2,431 bp, showed similarity with LOX genes from *Arachis* or other species in plant EST databases (http://www.ncbi.nlm.nih.gov/nucest/). Based on these sequence similarities, the sequences were assigned to three genes.

The sequences from clones C05 (GenBank accession number KP710973) and E10 (KP710974) (hereafter named *AhLOX6),* had a complete open reading frame (ORF) of 2,604 bp, a 68 bp 5'-UTR (untranslated region) and 166 bp of 3'-UTR. The gene encodes a predicted 867 amino acids with the estimated molecular weight of 96.785 kDa. Clones C05 and E10 had 97.1% nucleotide sequence identity overall and their deduced amino acid sequences were 100% identical. A GenBank search found this gene was most similar to *Glycine max LOX9* (gb|ABS32275.1, gene ID: 100127399) and *Phaseolus vulgaris* (common bean) lipoxygenase (gb|AAB18970.2, gene ID: 18618345) (Additional file 1). *Glycine max LOX9* was most highly expressed in mature nodules and in roots, where it specifically expressed in the developing phloem [35]. In nodules, the expression of *LOX9* was correlated with the development of cells in the vasculature and lenticels. Histochemical analyses suggested that *Glycine max* LOX9 is involved in the growth and development of specific cells within these tissues. The *Phaseolus vulgaris* (common bean) lipoxygenase was also highly expressed in young, developing nodules [36]. Gene structure prediction by aligning the sequence to *Glycine max LOX3* (emb|X06928.1, gene ID: 547869) showed nine exons and eight introns for *AhLOX6*.

For the second gene, *AhLOX7* (GenBank accession number KP710975)*,* two identical sequences (G02, H02) were most similar (82% identity) to *Glycine max* probable *linoleate 9S*-*lipoxygenase 5-like* mRNA (Gene ID: 100802887), *Glycine max LOX3* mRNA (U50081.1), which is highly expressed in seeds [35,37], and *Medicago truncatula* lipoxygenase mRNA (XM_003591072.1|) (Additional file 1). *AhLOX7* showed lower nucleotide similarity with *AhLOX2* and *AhLOX3*, 60% nucleotide sequence identity with *AhLOX4* (gb|EZ722311.1), and 97% with *AhLOX5* (gb|JR564445.1) with only three nucleotide differences in the ORF. The *AhLOX7* sequence has a complete open reading frame (ORF) of 2,583 bp, with a 90 bp 5'-UTR and 249 bp of 3'-UTR. The gene encodes 860 predicted amino acids with an estimated molecular weight of 96.961 kDa. The probable linoleate 9S-lipoxygenase 5 (UniGene Gma.24859, gene ID: 100802887) for *Glycine max* was predicted to be highly expressed in seed coat based on the EST profile from NCBI. The translated amino acid sequence showed 99.65% identity (Additional file 2) between *AhLOX7* and *AhLOX5*. Since peanut *LOX5* fell into the type I 9-LOX cluster, *AhLOX7* probably also belongs to the type I 9-LOXs [21]. Due to high predicted amino acid sequence identity, *AhLOX7* and *AhLOX5* may be different forms of the same gene, but given that *AhLOX2* and *AhLOX3* also had high identity between deduced amino acid sequences (99.42%) [20] we chose to assign a different name. This observation along with sequence variation among amplicons targeting *AhLOX7* described below (Additional file 3; Additional file 4), indicates that a more systematic study of peanut lipoxygenase genes in the peanut genome must be done to thoroughly characterize this multi-gene family. By aligning *AhLOX7* sequences with the soybean *LOX3* gene (emb|X06928.1 Gene ID: 547869), nine exons and eight introns were predicted. PCR spanning intron 1 indicated that the size of the first intron is greater than 3 kb, which is much larger than that in soybean *LOX3* (emb|X06928.1 Gene ID: 547869) (449 bp). The linoleate 9S-lipoxygenase 5-like sequence from soybean (gene ID: 100802887) has an intron 1 size of 1,956 bp, which is comparable with that of *AhLOX7*. Based on soybean lipoxygenase gene information [38,39], the corresponding locations of *AhLOX7* conserved histidine residues and fatty acid, iron, and oxygen binding sites were located in exons 7 to 9 (Table 1).

**Table 1:** **Corresponding amino acid residue positions of** ***Glycine max LOX3*** **and** ***Arachis hypogaea AhLOX7*** **for conserved amino acids**

**Amino acid conserved region for** ***Glycine max*** **LOX3** ^**1**^	**Fatty acid binding site**	**Iron binding site**						**Oxygen binding site**
**Amino acid abbreviation**	**(H)**	**(H)**	**(H)**	**(H)**	**(H)**	**(H)**	**(N)**	**( I)**
*Glycine max* seed LOX 3 (emb|X06928.1|)	513	518	523	541	550	709	713	857
*Glycine max* exon	E7	E7	E8	E8	E8	E9	E9	E9
*Arachis hypogaea AhLOX7*	516	521	526	544	553	713	717	860
*Arachis hypogaea* exon	E7	E7	E8	E8	E8	E9	E9	E9

^1^The conserved histidine residues, the fatty acid-, iron-, and oxygen binding- residue in *Glycine max* are according to Reinprecht et al. [38].

For the third gene, *AhLOX8,* four sequences (D03, E03, H07, and A09) were most similar to *Glycine max* lipoxygenase-10 (*LOX10*) mRNA (Additional file 1). Cloned insert sizes ranged from 1,821 to 2,431 bp. The longest sequence (H07) (GenBank accession number KP710976) had an open reading frame (ORF) of 2,214 bp. The *Glycine max LOX10* was reported to have a lower expression level than *LOX9* in leaf and nodules but a similar expression level in roots [37]. Gene structure prediction by aligning these sequences to soybean *LOX3* (emb|X06928.1 gene ID: 547869) showed nine exons and eight introns.

Our RT-PCR results (Additional file 5) indicated that *AhLOX6* was expressed in roots and leaves, *AhLOX7* was expressed in seeds, roots and leaves, while *AhLOX8* also was expressed in seeds, roots, and leaves, but probably at lower levels than *AhLOX7*. Since *AhLOX7* was expressed at high levels in roots and seeds, and putative soybean ortholog (UniGene Gma.24859) was predicted to be highly expressed in the seed coat, seed tissues being the sites of *Aspergillus* invasion, *AhLOX7* was targeted for mutation discovery. Furthermore, differential expression of a highly similar lipoxygenase (*AhLOX5*) in genotypes resistant or susceptible to *Aspergillus* infection recently was documented [21]. Additional file 6 shows the phylogenetic relationships among all described peanut lipoxygenase genes.

### Reference sequence amplification and characterization

For mutation detection, amplicon sequences from the genotype 'Tifrunner' (the wild type of the TILLING population) were used as references for alignment of short reads. Previously published peanut allergen genes *Ara h 1.01*, *Ara h 1.02*, *Ara h 2.01*, *Ara h 2.02*, and genes controlling oleic:linoleic acid ratio (O/L) - *ahFAD2A*, and *ahFAD2B* were also used in this study [40,41]. The “01” and “02” or “A” and “B” indicates that they are homeologous, with one copy in each subgenome.

For *AhLOX7* and *AhPLD*, the unique sequences obtained from amplicon sequencing were extracted as references. Because there was sequence variation for each gene from amplicon sequencing, more than one sequence per gene was used for reference (Additional file 3, 4, 7 and 8).

**Figure 1:**
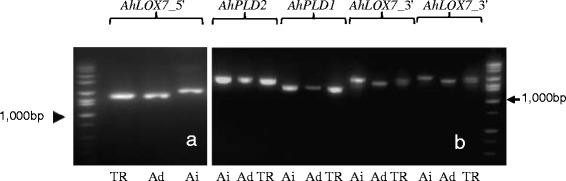
**PCR amplicons of** ***AhLOX7*** **_5',** ***AhLOX7_3*** **',** ***AhPLD1*** **, and** ***AhPLD2.*** PCR amplification of *AhLOX7_5*', *AhLOX7*_3', *AhPLD1*, and *AhPLD2*. **a**. Primers 2199/2184 amplify *AhLOX7_5*' on both progenitor genomes and the tetraploid. Amplicons cover partial E2, partial E4, entire E3, I2, and I3. **b**. Primers 1991/1992 amplified *AhPLD2* in tetraploid and diploid progenitor species; primers 2120/2121 amplified *AhPLD1* in both sub-genomes; primers 2187/2188 amplified *AhLOX7_3*' in both sub-genomes, amplicons contain partial E7, E9, entire E8, I7, and I8; primers 2186/2188 amplified *AhLOX7_3*' in both sub-genomes at a slightly different amplification start position resulted in similar amplification. DNA size standard: All-purpose Hi-Lo DNA marker, Bionexus, catalogue no. BN2050. TR = *A. hypogaea* cv. Tifrunner, Ad-*A. duranensis* (A genome), Ai = *A. ipaensis* (B genome).

Two primer sets were designed to amplify the 5' and 3' ends of *AhLOX7*, aimed at identifying mutations with early transcription truncation or at functionally critical domains (Tables 1 and 2). The 5' amplicons (Figure 1a) spanned exons 2 (partial), 3, and 4 (partial) while the 3' amplicon (Figure 1b) covered exons 7 (partial), 8, 9 (partial), and the corresponding introns. Sequencing of the 5' amplicon revealed at least four copies with only three nucleotide positions differing overall (Additional file 3). Six sequence groups were obtained from the 3' amplicon with lengths of 1,532 bp, 1,544 bp, 1,698 bp, and 1,710 bp. The sequences can be divided into five categories based on nucleotide similarities. Totally there were 32 variant sites within the 3' end amplified region (Additional file 4). Based on the high sequence similarity between *AhLOX5* and *AhLOX7* (Additional file 2), the amplicon used for TILLING might be a mixture of both genes.

**Table 2:** **Summary of amplicon sizes, mutation frequency, and primers used for the amplification**

**Gene**	**Amplicon (bp)**	**F primer no.**	**Sequence (5'-3')**	**R primer no.**	**Sequence (5'-3')**
*Ara h 1.01*	1,865	1306	GAGCAATGAGAGGGAGGGTT	2079	TCTTCGTCTTCGTCCTCCTCTTCTT
*Ara h 1.02*	1,666	1306	GAGCAATGAGAGGGAGGGTT	1309	CCTCCTCTTCTTCCCACTCTTG
*Ara h 2.01*	1,300	815	CGATTTACTCATGTACAATTAACAATAGAT	817	TCAAGATGGTTACAACTCTTGCAGCAACA
*Ara h 2.02*	1,247	816	ATCACCTTAAATTTATACATATTTTCGG	817	TCAAGATGGTTACAACTCTTGCAGCAACA
*Ah FAD2A*	1,241	1048	CTCTGACTATGCATCAG	1055	GATTACTGATTATTGACTT
*Ah FAD2B*	1,234	1048	CTCTGACTATGCATCAG	1101	CAGAACCATTAGCTTTG
*AhLOX7_5*'	1,713 1,714 1,715 1,716	2199	GCAGGAGAAGCAGCATTCACAGTTA	2184	CTCAAGAGGAACATTATCCC
*AhLOX7_3*'	1,532 1,544 1,698 1,710	2186	AAAGTCTACGGTGATCAAACCAGC	2188	AGCAGACACACCCATTGAAA
*AhPLD1*	1,271 1,272	2120	GACTTACGAACCTCAAAGATGCTGG	2121	TACTCTCCGTCCTTCTTCGCTT
*AhPLD2*	1,500	1991	AAGAACTGGGCACGTGGTGTTAGGAGT	1992	TCGACGGTTCTCCTGGGCTTTTATGTA

The amplification of *AhPLD1* at exon 2 indicated at least four sequence groups. An overall sixteen single nucleotide differences were detected. Groups 1, 2, and 4 had only one or two nucleotide differences between each other and group 3 was most different from other three (Additional file 7). Amplification of diploid progenitor species *A. duranensis* (A genome) and *A. ipaensis* (B genome) with the primer set designed for *AhPLD1* did not show genome specific amplification at these regions (Figure 1b).

The amplicon sequences of *AhPLD2* indicated at least five copies for *AhPLD2* exon 3. There were five nucleotide differences in 1,500 bp. Sub-genome specific amplification was not detected from diploid progenitor species using the same set of primers (Figure 1b; Additional file 8).

### Mutation detection, and validation for single copy genes

We obtained 367.8 million reads from one lane of an Illumina HiSeq run. After pre-processing, a total of 240 million reads (65%) remained. For the twenty libraries (12 from each column and 8 from each row), each library contained 3% to 7% of the total reads; the average read length was 91.44 bp. Over 85% of reads had the full read length of 94 bp.

The single copy genes included in this study were *Ara h 1.01*, *Ara h 1.02*, *Ara h 2.01*, *Ara h 2.02*, *AhFAD2A*, and *AhFAD2B*. The average depth of coverage for each sample for each gene per library at quality score cut-off from 10 to 25 was 76.01 (average depth of coverage for each sample for each gene across libraries = the total depth of coverage/(the number of genes × number of libraries × number of samples)). The average depth of coverage per sample for each gene per library was 76.5 at sequence quality score 10 and 75.2 at quality score 25. The average depth of coverage for each sample varied for different genes and libraries. The non-reference base percentage (percentage of reads containing a SNP compared with the reference sequence) was calculated for all six known mutants with sequence quality scores ranging from 10 to 21 to set up mutation detection parameters (Table 3; Additional file 9). Under these parameters (sequence quality scores from 10 to 21, and minimum read percentage with non-reference nucleotide from 0.058% to 0.37%), a set of unique mutants was detected (Table 4). We found that when the quality score was increased, the minimum read percentage with non-reference nucleotide had to be decreased to detect all six know mutations. However, with the combination of higher sequence quality score and lower minimum non-reference percentage, the number of predicted mutations increased. So, changing the minimum read percentage with non-reference nucleotide could be critical to control false positive predictions. When the sequence quality score increased to 22 and above, no known mutants could be detected, possibly due to a less than average number of mutations in the libraries caused by either uneven pooling and/or amplification efficiency. Because our purpose was to search for unknown single nucleotide mutations over a set of known sequences (amplicons) within a certain frequency range, the mutations that could be consistently detected across all sequence qualities (even at lower sequence quality cut-offs) was less likely due to sequencing errors.

**Table 3:** **Mutation prediction parameters in various ranges for single copy genes**

**Min quality**	**Minimum read percentage with non-reference nucleotide** ^**1**^ **(%)**	**Background read percentage with non-reference nucleotide** ^**1**^ **(%)**	**Maximum read percentage with non-reference nucleotide (%)**	**Row non-reference % multiplier**	**All mutants (uniquely found** ^**2**^ **)**	**Known mutants (uniquely found** ^**2**^ **)**	**No. of new mutations validated**
10	0.370	0.033	5.000	0.67	33	6	9
12	0.370	0.030	5.000	0.67	34	6	8
14	0.370	0.030	5.000	0.67	34	6	8
15	0.370	0.029	5.000	0.67	32	6	8
16	0.360	0.029	5.000	0.67	31	6	9
17	0.190	0.026	5.000	0.67	55	6	12
18	0.170	0.023	5.000	0.67	54	6	13
19	0.170	0.020	5.000	0.67	52	6	13
20	0.135	0.018	5.000	0.67	71	6	12
21	0.058	0.017	5.000	0.67	355	3	7

^1^The average non-reference nucleotide percentage at each quality cut-off.

^2^The mutations were only found once in each row and column.

**Table 4:** **Summary of mutations identified in this study**

**Gene**	**Nucleotide change**	**Predicted AA change**	**Population**	**Plant ID**	**Amplicon length**	**SIFT score**
*Arah1.01*	C321 → T	Silent	08 F	213_1	1,865	
*Arah1.01*	C1524 → T	T377 → I	07JKEMS1	67	1,865	0.11
*Arah1.01*	C 1678 → T	Silent	07JKEMS1	48	1,865	
*Arah1.02*	A 1258 → G	silent	07JKEMS1	125	1,666	
*Arah1.02*	G72 → T	Q 24 → H	07JKEMS1	125	1,666	0.12
*Arah1.02*	C428 → T	P143 → L	08 F	216_1	1,666	0.16
*Arah1.02*	C 644 → A	P215 → H	08 F	221_5	1,666	0.00
*Arah1.02*	C765 → T	Silent	07JKEMS1	125	1,666	
*Arah1.02*	G 891 → A	Silent	07JKEMS1	2	1,666	
*Arah1.02*	A694 → G	I232 → V	07JKEMS1	125	1,666	1.00
*Arah1.02*	A 742 → C	K248 → Q	07JKEMS1	125	1,666	0.47
*AhFAD2B*	C 632 → T	P 211 → L	08 F	222_3	1,234	0.00
*Arah2.02*	C → T (upstream)	Silent	08 F	231_4	1,247	
*AhLOX7_3*'	T 1508 → C	L503 → P	07JKEMS1	69	1,532	0.00
*AhLOX7_5*'	C512 → G	A171 → G	07JKEMS1	125	1,714	0.09
*AhLOX7_5*'	A525 → G	I 175 → M	07JKEMS1	125	1,714	0.07
*AhLOX7_5*'	C532 → G	L178 → V	07JKEMS1	125	1,714	1.00
*AhPLD1*	C1328 → T	S 443 → F	08 F	201_4	1,272	0.00
*AhPLD1*	G1632 → A	M 544 → I	08 F	211_5	1,272	0.01
*AhPLD2*	C1727 → T	P 576 → L	07JKEMS	67	1,500	0.00

In theory, if the amplicons were mixed at equal quantity, in the row pool, the expected read percentage with non-reference nucleotide should be 1.04% (1/96 × 100%) for homozygous mutants and 0.52% (1/192 × 100%) for heterozygous mutants, while in the column pool, the non-reference reads should account for 1.56% (1/64 × 100%) and 0.78% (1/128 × 100%) for the homozygous and heterozygous mutants, respectively. The read percentage with non-reference nucleotide can be lower than the theoretical value because of sequencing errors, uneven amplicon pooling, etc. Decreasing the quality score can result in false-positive mutants due to sequencing errors (type I error), while increasing the quality score will decrease the mutation detection sensitivity (type II error). Thus the known mutants became very important for selecting the appropriate parameters that were sensitive enough to detect non-reference nucleotides and reliable enough to distinguish them from sequencing errors. When unique mutants were detected, all other nucleotides under the hypothetical mutation positions were also calculated; the hypothetical mutations should have significantly greater numbers of mutation-responsible nucleotides than other non-reference nucleotides. We found that the minimum read percentage with non-reference nucleotide needed to predict a mutation was 3–12 times the average non-reference nucleotide frequency.

With the above parameters, 14 mutants were found at all quality scores (“common group”), 34 detected only at sequence quality scores 17 to 20 (“high group”), and 15 detected at sequence quality scores 10 to 16 (“low group”), respectively. Subsequent mutation validation was done by several methods. Initially, CAPS (Cleaved Amplified Polymorphic Sequences) assay was carried out for all predicted mutations that were amenable. From the “common” mutation group, six were suitable for CAPS assay (three *Ara h 1.01* and three *Ara h 1.02*), and five were validated (two *Ara h 1.01* and three *Ara h 1.02*). From the "high" group, 13 were appropriate (two *AhFAD2B*, one *Ara h 1.01*, five *Ara h 1.02*, three *Ara h 2.01* and two *Ara h 2.02*), although only two *Ara h 1.02* mutations were confirmed. From the “low” group, seven putative mutants were screened (one *AhFAD2A*, one *AhFAD2B*, two *Ara h 1.02*, two *Ara h 2.02*, and one *Ara h 2.01*), but none of them was validated. Since the “common” group had the highest validation rate, SSCP (Single Strand Conformational Polymorphism) was then carried out for the remaining nine samples in this group; only one was validated. Because SSCP might have a higher false-negative rate, these nine samples were then tested by amplicon sequencing and four mutations were detected, including the previous one identified by SSCP. This might indicate that SSCP was not sufficiently sensitive for mutation detection in our study. Altogether, a total of 11 predicted mutations were confirmed by the combined CAPS/SSCP/amplicon sequencing approach.

For the remaining twenty samples in the “high” group and seven samples in the “low” group, five of them were predicted to have missense mutations (three from the “high” group and two from the “low” group) by Geneious software (http://www.geneious.com/) and thus were selected for amplicon sequencing. Two from the “high” group were validated. Altogether, 13 mutations were identified in the single copy genes. Comparing the validation results with the mutation detection parameters, optimum parameters of phred score = 18 or 19, with the minimum read percentage with non-reference nucleotide of 0.17% and maximum read percentage with non-reference nucleotide of 5% could predict the largest number of “true” mutations (Table 3).

In this experiment, we included 384 individuals from our previous TILLING study. In addition to the six previously identified mutants, eight more mutants were recognized. Five additional mutants were identified from the four new TILLING plates (384 individuals). This indicated that TILLING by sequencing is a more sensitive method for mutation detection than the CEL I/LI-COR heteroduplex detection method. One of the possible reasons is that for LI-COR gels, 200 bp are excluded from the amplicon to adjust for the 100 bp regions at the top and bottom of TILLING gel images that are difficult to analyse, which will shorten the actual TILLING target region. Another reason could be that CEL I preferentially recognizes certain mismatches, C/C ≥ C/A ~ C/T ≥ G/G, over others (A/C ~ A/A ~ T/C > T/G ~ G/T ~ G/A ~ A/G) which might decrease the sensitivity for detecting these mutations [42,43].

Three mutations were identified in *Ara h 1.01*. The C to T transition at bp positions 321 and 1687 are silent (Table 4). The third predicts a T 377 I change. The substitution was predicted to be tolerated according to SIFT analysis (http://sift.jcvi.org/) [44] (Table 4). Eight mutations were identified in *Ara h 1.02*. Three of the mutations, with a C to T transition at bp position 765, an A to G transition at bp position 1,258, and a G to A transition at bp position 891, are silent. The other five are predicted to induce amino acid changes: Q 24 H, P 143 I, P 215 H, I 232 V, and K 248 Q. The proline to isoleucine change at position 143 lies within epitopes 8 and 9 [45]. The proline to histidine change at position 215 was predicted to affect protein function by SIFT analysis (p < 0.05). Line 125 had five nucleotide changes in *Ara h 1.02*, two of which were silent.

Only known mutations were re-detected in *Ara h 2.01* and *AhFAD2A*. A new mutation was identified in *Ara h 2.02*. The C to T mutation was found upstream of the start codon, but doesn’t appear to be located within any important promoter regions. One predicted amino acid change (P 211 L) that might affect protein function according to SIFT analysis was found in *AhFAD2B* (Table 4).

There are two possible reasons the predicted mutations were not validated. Firstly, because homeologous genes were used as TILLING references, in theory, the mutations predicted at identical regions for homeologous genes should be reported for both genes, while most of the time mutations from only one gene were reported due to the default BWA settings, which caused bias in the subsequent validation. Secondly, the false positives could arise from sequencing errors introduced by the combination of lower quality reads (especially for the mutations identified from the “low group”) and the ambiguous mutation detection threshold. The minimum read percentage with non-reference nucleotide is set universally for each gene and each library once the quality cut-off is set, while the sequence depth for each gene and library may vary so that for a certain genes or libraries the true mutation calling may be mixed with noise.

### Mutation prediction and validation in multi-copy genes

Seventeen validated mutations (eleven of those newly discovered from above and six known) were used as internal positive controls to detect mutations for multi-copy genes, with two major improvements. Firstly, the true mutation percentage and false mutation percentage for the reference genes at all quality scores were calculated and were used as reference to set the mutation detection parameter (Table 5). The improvement was made because when analyzing the first six known mutants, several mutants were relatively easier to detect, whereas one or two others were more difficult to detect. The more cryptic mutants required more relaxed parameters. These relaxed parameters introduced more noise into the results, and likely increased false positives. When using a larger set of reference mutations, and with multiple highly similar sequences, the problem of finding a relaxed value for read percentage with non-reference nucleotide that allowed detection of the entire mutation set was amplified. We thus updated our mutation detection strategy by maximizing efficiency (fewer false and unknown mutants) at the cost of decreasing the total number of true mutants. Secondly, a true/false mutant factor was added for mutation identification. While looking through the results for true mutants missed from the single copy gene prediction, we found that most missed mutants were typically counted ~10-20 times more in 1 row/column, when compared to variant counts in other rows and columns; however, the variant percentage was below the specified minimum non-reference percentage. Thus using minimum variance multiplier will be a more sensitive method for mutation prediction with multi-copy sequences. In addition, in Bowtie 2 alignments, options were set to report all alignments instead of in the previous experiment when the BWA default parameters were used (report first three alignments). This approach was taken because mutations detected in a conserved region for multi-copy genes could not be assigned to a specific copy without further validation (Table 5).

**Table 5:** **The top ten mutant prediction parameters that produced the highest percent of true mutant and false mutant percentage difference using the known mutants as control**

**Minimum quality score**	**Minimum variance multiplier**	**Minimum variance percentage**	**Number of true mutants** ^**1**^	**True %**	**Number of false mutants**	**False %**	**Total mutants predicted**	**Difference**
19	12	0.05	11	68.8	1	6.2	16	62.6
19	14	0.05	10	66.7	1	6.7	15	60.0
20	12	0.05	12	63.2	1	5.3	19	57.9
21	12	0.05	12	63.2	1	5.3	19	57.9
16	10	0.05	11	68.8	2	12.5	16	56.3
22	10	0.05	11	61.1	1	5.6	18	55.5
23	8	0.05	11	61.1	1	5.6	18	55.5
21	10	0.05	12	60	1	5	20	55.0
15	10	0.05	10	66.7	2	13.3	15	53.4
16	12	0.05	10	66.7	2	13.3	15	53.4

^1^Based on the validated mutants and the known mutants detected from previous study [40].

For *AhLOX7*, seven out of the top ten predicted mutations had minimum quality scores above 19, and the minimum variance multipliers around 12. We selected six sets (minimum quality 19, 20, 21, with minimum variance multipliers 12 and 14, and the minimum read percentage with non-reference nucleotide of 0.05%) as candidate mutants. Seven of the hypothetical mutants were predicted to be missense and were chosen for validation, including one *AhLOX7*_3’ and six *AhLOX7*_5’. The *AhLOX7*_3’ and three *AhLOX7*_5’ were validated by CAPS assay with expected digestion patterns. The *AhLOX7*_3' accounts for a leucine to proline change at bp position 1508. This mutation is located in exon 7, but not at the fatty acid binding site. The SIFT (Sorting Intolerant from Tolerant) prediction [44] indicated this amino acid change will affect protein function (p < 0.05). All three mutations at the 5' end of *AhLOX7*, A 17 G, I 175 M, and L178 V, were found in exon 2 and in line 125 (Table 4). These amino acid changes were less likely to affect protein function according to SIFT (p < 0.05).

For *AhPLD1* and *AhPLD2*, mutations were calculated with minimum quality scores from 10 to 25, and minimum variance multipliers from 2 to 20. Results showed the increase of minimum variance multiplier decreased the number of predicted mutations, as expected, while the increase of minimum quality had little effect on the predicted mutation numbers, which ranged from 4 to 40 when the parameters were changed. We chose twelve sets of parameters to keep the number of predicted mutations within a reasonable range, namely a minimum variance multiplier of 12 and 14 at quality 19, and minimum variance multipliers of 6, 8, 10, 12, and 14 at sequence quality of 20 and 21. All three mutations (all were missense) that were common to all parameter sets were selected for validation. Both sequencing and CAPS assay confirmed that all three predicted mutants were real. Predicted amino acid changes are serine to phenylalanine at position 433, methionine to isoleucine at position 544 for *AhPLD1* and proline to leucine at amino acid 576 for *AhPLD2*. The substitution at position 443 from S to F and the substitution at position 544 from M to I for *AhPLD1* and the substitution at position 576 for *AhPLD2* were predicted to affect protein function according to SIFT. None of them were located in the PLD active site HxKxxxxD (HKD) motif [46], or at the Ca^2+^-dependent phospholipid binding C2 domain [46-48].

In total, 8.5 kb of the peanut genome was screened for mutations in single copy genes and 27.6 kb was screened for multi-copy genes. Twenty induced point mutations were identified. Single nucleotide substitution was identified in both coding and non-coding regions. Among the identified mutations, six were silent and fourteen were missense mutations; no nonsense mutations were found. The silent/missense mutation ratio is biased because we focused on validating non-silent mutations after the initial CAPS assay for single copy genes. The mutation frequency was calculated as follows: the size in base pairs of the region screened was multiplied by the total number of lines screened per total number of identified mutations. Thus, the overall mutation frequency was 1 SNP/ 1,066 kb ([(8.5 + 27.6)/ (20 + 6)] × 96 × 8). A total of thirteen mutations were newly identified for single copy genes in this study. The SNP frequency for single copy genes was 1 SNP/344 kb ([8.5/19] × 96 × 8) and the SNP frequency for multi-copy genes was 1 SNP/3,028 kb ([27.6/7] × 96 × 8). Our TILLING population was treated with 0.4% EMS for 12 hours, and compared with the previously reported mutation frequency of 1 SNP/1,067 kb for single copy genes for the same population, the current TILLING by sequencing method had a higher chance for mutation detection [40]. Eleven out of twenty nucleotide changes were G to A or C to T transitions, as expected for EMS-induced mutations. The other nine were unusual mutations. When looking closely at the unusual mutation types, eight out of nine were from the 07JKEMS1 population and out of these seven were from line 125. This line also had more than one base change detected in amplicons, for multiple genes. Moreover, some of the mutations were typical EMS-induced G/C to A/T transitions and others were not. All mutations in line 125 were heterozygous. Therefore line 125 might be interpreted as a suspicious contaminant (except for the high heterozygosity rate). If this sample is excluded, the mutation frequency would be 1 SNP/ 1,459 kb ([(8.5 + 27.6)/(26–7)] × 96 × 8) overall, and 1 SNP/466 kb ([8.5/(19–5)] × 96 × 8) for single copy genes, still higher than the previous report, and 1 SNP/5,299 kb ((27.6/4) × 96 × 8) for multi-copy genes. The mutation frequency decreased from multi-copy gene screening because multi-copy genes are highly similar in sequence and the short reads mapped couldn’t be separated to a specific copy. Thus the multi-copy genes have a lower read percentage with non-reference nucleotide than single copy genes. For mutations in the conserved region to be effectively detected, perhaps a higher sequencing depth/minimum read percentage with non-reference nucleotide coverage needs to be achieved. Besides, thresholds need to be set to distinguish gene specific nucleotide differences from real mutations. By comparing with the previous TILLING by sequencing study [17], our mutation detection method has several differences. Firstly, we used a set of known mutations to adjust the parameters for mutation detection. When all known mutations can be detected, we set the threshold for minimum and maximum read percentage with non-reference nucleotide to detect new mutations for single copy genes. Secondly, we developed a method for detecting mutations in multi-copy, highly similar genes. After validating the single copy genes, our mutation detection pipeline was improved by setting the mutations at the ratio of non-reference nucleotide number counts of rows to columns at each position (variant multipliers), which ranged from 8 to 14 at the minimum non-reference nucleotide percentage of 0.05% and Phred quality scores ranging from 16 to 23. Although this method won’t allow us to detect the exact gene in which the mutation resides for the highly conserved regions, the subsequent mutation validation showed it was effective in discerning real mutations from background in spite of gene copy number. Thirdly, due to the fact that mutation validation is time consuming, especially for multi-copy genes, and that the uneven pooling and PCR efficiency will all cause some mutations to be easier to detect than others, our mutation detection strategy focused on efficiency instead of detecting all possible mutations. After validating all the mutations, a few of the mutations were found to have a higher minimum non-reference coverage or a lower minimum non-reference coverage than the majority of the other known mutations. Those mutations will obscure the threshold and allow more false positive mutations to be predicted, thus will complicate the subsequent validation process. The updated pipeline set a threshold where the majority of mutations could be detected, which improved the efficiency.

## Conclusions

By combining sequence quality score with minimum read percentage with non-reference nucleotide or non-reference variance multiplier, with the control being previously validated mutations, we refined our mutation discovery pipeline to identify mutations in both single copy and multiple copy genes. TILLING by sequencing identified previously reported genes as well as new mutations from the same population. We also provide evidence that the *AhLOX7* and *AhPLD* genes have multiple copies in the peanut genome. The mutations identified can be used to further study gene function.

## Methods

### cDNA library screening and probe design

A cDNA library was constructed from developing roots of peanut (*Arachis hypogaea* L.) genotype C724-19-25 [49] (the isogenic line of TifGuard) with the In-Fusion®SMARTer_®_ directional cDNA library construction kit (Clontech, Mountain View, CA) following the user’s manual. Probes were designed from a conserved region identified by aligning EST sequences retrieved from GenBank by similarity to *AhLOX1-3* using the Align X feature with Vector NTI®Suite V6.0 (InforMax, Bethesda, MD). The probes were amplified by PCR using TifGuard as the template, with the amplicon sizes ranging from 150 bp to 300 bp. PCR products were purified with QIAquick Gel extraction kit (Qiagen Inc. Valencia, CA, USA), then were mixed in equal amounts for library screening. The cDNA library was plated on ten 150 × 15 mm Petri dishes (Fisher Scientific, Pittsburgh, PA), with around 2 × 10^4^ colonies per plate. The colonies were transferred to Genescreen Plus nylon membrane (Perkin-Elmer, Boston, MA) following Sambrook and Russell [50]. The probes were labelled with α^32^P-dCTP using the Random Primed DNA labelling kit (Roche, Indianapolis, IN). Unincorporated label was removed using Sephadex G-50 (Sigma, St. Louis, MO). Hybridization and washing followed Sambrook and Russell [50]. Hybridization signals were detected by exposing filters to film (X-U, Fuji Film, Tokyo). The corresponding clones were picked and plated for a secondary screening following the same protocol. Plasmid DNAs from colonies were cut with *Sac*I and *Xba*I (NEB catalogue No. R0156S, and R0145S) or *Eco*RI + *Bam*HI (NEB catalogue No. R010s and R0136s) to investigate the insertion sizes. Single clones with an insertion size > 2 kb were fully sequenced by primer walking at the Georgia Genomics Facility at the University of Georgia. RT-PCR was carried out by Superscript III first strand synthesis system for RT-PCR (Invitrogen, Carlsbad, CA).

The identified candidate LOX sequence that showed high expression in seeds was used for TILLING. Two amplicons of LOX were used for TILLING, one at the 5' end, starting at the second exon and ending at the fourth exon, and a second at the 3' end starting at exon 7 and ending at exon 9.

### Amplification of phospholipase D genes

In peanut, two types of full-length cDNAs were reported, each encoding distinct PLD molecules with 794 (*AhPLD1*) and 807 (*AhPLD2*) amino acid residues, respectively [33,34,51]. Peanut PLD sequences from the above published sources were collected and sorted according to their sequence similarities. Intron-exon prediction was carried out by Splign (http://www.ncbi.nlm.nih.gov/sutils/splign/splign.cgi?textpage=online&level=form). *AhPLD1* has three exons and two introns, while *AhPLD2* has four exons and three introns. Domain prediction by InterProScan indicated three domains for both PLD1 and PLD2. The TILLING primers for *AhPLD1* and *AhPLD2* were designed by CODDLE (http://www.proweb.org/coddle/; The link is no longer available after Dec 31, 2013, suggest primer design with the following link http://tilling.ucdavis.edu/index.php/Primer_Design_and_Testing_Guide). The amplicons were on the second exon for PLD1 and third exon for PLD2, respectively.

Amplicons from *AhPLD1*, *AhPLD2*, and *AhLOX7* were cloned with Zero-Blunt PCR cloning kit (Life Technologies, catalog no. K 2700–20) and colonies were sent to the Georgia Genomics Facility for Sanger sequencing. Sequences were analysed by Geneious software (http://www.geneious.com/).

### Illumina library preparation and multiplexing

The mutagenized peanut population development and genomic DNA extraction from individual M_2_ plants were previously described [40]. Equal amounts of DNAs were pooled following the bidimensional pooling strategy from Tsai et al. [17]. Briefly, eight 96-well plates were pooled into one 96-well plate according to their corresponding positions (micropools). Subsequently, 20 superpools were generated by combining all the wells in a row (a superpool containing 96 individuals) or in a column (a superpool containing 64 individuals) from the micropool. Primers for amplification of *Ara h 1*, *Ara h 2*, *AhFAD2A* and *AhFAD2B* genes were as described by Knoll et al. [40]. The PCR was carried out in a 20 μl final volume containing 20–30 ng gDNA, 0.4U iProof high fidelity DNA polymerase in 1 × PCR buffer (Bio-Rad, Hercules, CA), 0.2 μM each of dNTP, and 0.5 μM each forward and reverse primers. Reactions were conducted using a Gene Amp 9700 (Applied Biosystems, Carlsbad, CA) thermal cycler. The PCR conditions were as follows: denaturation at 98°C for 30 sec, then 30 to 35 cycles of 98°C for 10 sec, 61 to 65°C for 20 sec, and 72°C for 45 sec to 1 min.

All ten TILLING targets (Table 2) were amplified from the 20 superpools. PCR products were purified with QIAquick PCR purification kit (Qiagen, Valencia, CA) or Agencourt AMPure XP (Beckman Coulter, A63880), and then quantified using Quant-It PicoGreen dsDNA kit (Invitrogen, Carlsbad, CA). Amplicons from the same superpool were mixed for Illumina library preparation. Altogether, twenty five-base barcoded adapters were synthesized for Illumina sequencing [17]. The pooled amplicons were fragmented with NEBNext dsDNA fragmentase (NEB, catalog no. M0348S). Ends were repaired with NEB End Repair Module (catalog no. E6050S). The A bases were added to the 3'-end by NEB Klenow Fragment (NEB, catalog no. M0212s). Ligation was performed by NEB Quick Ligation kit (catalog no. M2200s). The size selection was done by QIAquick gel extraction kit (QIAGEN, catalog no. 287060). Products were enriched by PCR with Phusion High-Fidelity DNA polymerase (NEB, catalogue No. M0530s).

### Mutation detection bioinformatics pipeline

The raw sequence was de-multiplexed and quality filtered by the TILLING pipeline from the Comai lab (http://comailab.genomecenter.ucdavis.edu/index.php/TILLING_by_Sequencing) with modifications. A custom Python script was written to replace the Ns in barcodes by checking their mate pair’s barcode.

The analysis of single copy genes (*Ara h 1.02*, *Ara h 1.02*, *Ara h 2.01*, *Ara h 2.02*, *AhFAD2A*, *AhFAD2B*) was done by aligning the de-multiplexed sequences to reference sequences with BWA (Burrows-Wheeler Aligner) [52] using parameters –q 20 –k 1, which follows the parameter from the TILLING pipeline from the Comai lab. Samtools was used for creating Mpileup files for each library [53]. A custom Python script was written to pre-process the Mpileup files to create filtered pileups at the minimum Phred quality scores of 10, 12, 14, 15, 16, 17, 18, 19, and 20 for each alignment. The number of unique mutants was counted by a custom Python script when the maximum read percentage with non-reference nucleotide was 5%, and minimum read percentage with non-reference nucleotide ranged from 0.135% to 0.37%. Since the column super-pool had 2/3 the number of samples of the row super-pool, a dilution factor of 0.67 was used on non-reference percentages for column libraries when comparing with row libraries. Six known mutations from a previous study were used as internal controls to ensure that all six mutants were detected at all parameter sets selected and to determine the most efficient mutant calling parameters [40]. Unique mutants were filtered out from the mutant list for further validation. Mutants retrieved using the above parameters were compared and grouped into three categories according to the Phred quality score at which they were identified. Mutants found at all quality scores were placed in a “common” group, mutants found only at Phred scores 10 to 16 were in the “low” group, and mutants found only at Phred scores 17 to 20 were in the “high” group.

After validating the single copy genes, all validated mutants along with the six previously detected mutations were again used as internal controls for setting the parameters to detect multi-copy genes. The updated multi-copy gene mutation detection pipeline aligned all Illumina reads to reference sequences with Bowtie2, which can perform local alignments and exclude ends with low quality or similarity [54], with the parameters set as: perform local alignments allowing 1 seed alignment mismatch and report all alignments. Then the minimum variant percentage values were used to determine the cut-off value of a real mutant. The updated pipeline filtered mutants based on relative variance at the ratio of alternative nucleotide number counts of rows to columns at each position (variant multipliers), which ranged from 8 to 14 at the minimum non-reference count percentage of 0.05% and Phred quality scores ranging from 16 to 23 with a step of 1. With the updated known mutants as references, the numbers of known mutants detected and undetected were reported, respectively. By calculating the percentage of known true and false mutants, parameters resulting in the highest true mutation detection percentage and the lowest false mutation detection percentage were given priority; candidates detected from the above mentioned parameters were used for further validation.

### Mutant validation

All the candidate mutants were analysed by Geneious for potential amino acid changes and CAPS designer (http://solgenomics.net/tools/caps_designer/caps_input.pl) for their capability of CAPS assay. For multi-copy genes, only restriction enzymes with digestion patterns unique to the mutant gene were suitable for CAPS assay. The amplicons were purified by QIAquick PCR purification kit (Qiagen Inc. Valencia, CA, USA) or Agencourt AMPure XP (Beckman Coulter Inc A 63880), and digested with corresponding restriction enzymes. The digested fragments were separated by Ultra-Pure Agarose (Invitrogen) or NuSieve GTG agarose (Lonza Rockland, Atlanta, GA).

For samples not suitable for CAPS assay, SSCP was carried out for validation following the protocol of Zeng et al. [40]. Mutants identified from CAPS assay and SSCP were further confirmed by Sanger sequencing of amplicons (single copy genes) or performing PCR cloning with Zero-Blunt PCR cloning kit (Life Technologies, catalogue no. K 2700–20) prior to sequencing. The mutation effect was analysed by SIFT (Sorting Intolerant from Tolerant, http://sift.jcvi.org/) with default parameters [44]. Amino acids with probabilities <0.05 are predicted to affect protein function.

## Additional files

Additional file 1: Table S1. GenBank search results for the newly discovered LOX genes. Additional file 2: Table S2. Percent identity matrix from deduced amino acid sequences. Additional file 3: Table S3. Sequence variation of *AhLOX7_5*' amplicon. Additional file 4: Figure S13 Alignment of all unique sequences from *AhLOX7* 3’ amplification (2186/2187) demonstrates sequence variation. Additional file 5: Figure S2. Expression level of LOX genes in seeds and roots from RT-PCR. Additional file 6: Figure S3. Phylogenetic analysis of all LOX genes with EST sequences. The tree was displayed in topology layout; branch labels denote lengths. Additional file 7: Table S4. Sequence variation of *AhPLD1* amplicon. Additional file 8: Table S5. Sequence variation of *AhPLD2* amplicon. Additional file 9: Table S6. Mutations identified from previous study.
