# Supplementary material for: TILLING by sequencing to identify induced mutations in stress resistance genes of peanut (Arachis hypogaea)
Source: BMC Genomics. 2015 Mar 7;16(1):157. doi: 10.1186/s12864-015-1348-0 (PMC4369367; doi:10.1186/s12864-015-1348-0)
Supplement: Additional file 1: Table S1. — GenBank search results for the newly discovered LOX genes. [file 12864_2015_1348_MOESM1_ESM.docx]

## Table S1- GenBank search results for the newly discovered LOX genes

| Gene name | Sequence name | Nucleotide hit | Percent of identity | | Amino acid hit | Percentage of similarity | Percentage of identity | *AhLOX1* (gb\|AF231454.1) | *AhLOX2* (gb\|DQ068249.1) | *AhLOX3* (gb\|DQ068250.1) | AhLOX4  (gb\|EZ722311.1) | AhLOX5  (gb\|JR564445.1) |
| --- | --- | --- | --- | --- | --- | --- | --- | --- | --- | --- | --- | --- |
| AhLOX6 | C5 and E10 | *Glycine* *max* *LOX9* mRNA ([gb\|EU003576.1](http://www.ncbi.nlm.nih.gov/nucleotide/152926331?report=genbank&log$=nuclalign&blast_rank=2&RID=W5SMSYS301N)) | | 80% | *Glycine max LOX9* (gb\|ABS32275.1) | 87% | 78% | 67% | 69% | 69% | 66% | 58% |
|  |  |  | |  | *Phaseolus* *vulgaris* (common bean) lipoxygenase (gb\|AAB18970.2) | 88% | 78% |  |  |  |  |  |
| *AhLOX7* | G02 and H02 | *Glycine* *max* probable *linoleate* *9S*-*lipoxygenase* *5-like* mRNA (Gene ID: 100802887) | | 82% | linoleate 9S-lipoxygenase 5-like gene in *Glycine* *max* | 81% |  | 58% | 59% | 78% | 60% | 97% |
|  |  | *Glycine* *max LOX3* (U50081.1) | | 78% |  |  |  |  |  |  |  |  |
|  |  | *Medicago* *truncatula* lipoxygenase mRNA ([ref\|XM_003591072.1\|](http://www.ncbi.nlm.nih.gov/nucleotide/357441684?report=genbank&log$=nuclalign&blast_rank=3&RID=HKTXCENE014)) | | 78% | lipoxygenase gene in *Medicago truncatula* (XP_003591120.1) | 78% |  |  |  |  |  |  |
|  |  | *Malus* × *domestica* clone 5 lipoxygenase (*LOX1d)* mRNA ([gb\|KC706482.1\|](http://www.ncbi.nlm.nih.gov/nucleotide/485451112?report=genbank&log$=nuclalign&blast_rank=7&RID=HKTXCENE014)) | | 74% | *Corylus* *avellana* ([AJ417975.1](http://www.ncbi.nlm.nih.gov/nuccore/16904542)) | 74% |  |  |  |  |  |  |
| *AhLOX8* | D03, E03, H07, and A09 | *Glycine* *max* lipoxygenase-10 (*LOX10*) mRNA ([gb\|EU003577.1\|](http://www.ncbi.nlm.nih.gov/nucleotide/152926333?report=genbank&log$=nuclalign&blast_rank=1&RID=W5SMSYS301N)) | | 80-89% | *Glycine max* lipoxygenase-10 (gb\|ABS32276.1\|), | 82% | 90% | 59% | 60% | 58% | 66% | 54% |
