# Supplementary material for: TILLING by sequencing to identify induced mutations in stress resistance genes of peanut (Arachis hypogaea)
Source: BMC Genomics. 2015 Mar 7;16(1):157. doi: 10.1186/s12864-015-1348-0 (PMC4369367; doi:10.1186/s12864-015-1348-0)
Supplement: Additional file 2: Table S2. — Percent identity matrix from deduced amino acid sequences. [file 12864_2015_1348_MOESM2_ESM.docx]

## Table S2- Percent identity matrix from deduced amino acid sequences

|  | gi\|298114570 (LOX4) | H07  (LOX8) | E10  (LOX6) | C05  (LOX6) | gi\|68161355 (LOX2) | gi\|68161357 (LOX3) | gi\|7331094 (LOX1) | G02  (LOX7) | H02  (LOX7) | gi\|372416992 (LOX5) |  |
| --- | --- | --- | --- | --- | --- | --- | --- | --- | --- | --- | --- |
| gi\|298114570 (LOX4) |  |  |  |  |  |  |  |  |  |  |  |
| H07 (LOX8) | 73.91 |  |  |  |  |  |  |  |  |  |  |
| E10 (LOX6) | 67.25 | 67.94 |  |  |  |  |  |  |  |  |  |
| C05 (LOX6) | 67.25 | 67.94 | 99.77 |  |  |  |  |  |  |  |  |
| gi\|68161355 (LOX2) | 69.63 | 67.39 | 69.73 | 69.85 |  |  |  |  |  |  |  |
| gi\|68161357 (LOX3) | 69.28 | 67.39 | 69.5 | 69.62 | 99.42 |  |  |  |  |  |  |
| gi\|7331094 (LOX1) | 65.12 | 63.47 | 65.68 | 65.8 | 93.29 | 93.65 |  |  |  |  |  |
| G02 (LOX7) | 60.64 | 62.6 | 59.33 | 59.45 | 61.34 | 60.98 | 57.21 |  |  |  |  |
| H02 (LOX7) | 60.64 | 62.6 | 59.33 | 59.45 | 61.34 | 60.98 | 57.21 | 100 |  |  |  |
| gi\|372416992 (LOX5) | 61 | 62.6 | 59.57 | 59.69 | 61.58 | 61.22 | 57.45 | 99.65 | 99.65 |  |  |
