# Supplementary material for: TILLING by sequencing to identify induced mutations in stress resistance genes of peanut (Arachis hypogaea)
Source: BMC Genomics. 2015 Mar 7;16(1):157. doi: 10.1186/s12864-015-1348-0 (PMC4369367; doi:10.1186/s12864-015-1348-0)
Supplement: Additional file 3: Table S3. — Sequence variation of AhLOX7_5' amplicon. [file 12864_2015_1348_MOESM3_ESM.docx]

## Table S3 - Sequence variation of *AhLOX7_5*' amplicon

| Name | Length (bp) | 461^1^ | 684 | 1384 | Category |
| --- | --- | --- | --- | --- | --- |
| *AhLOX7_G1* | 1,715 | - | A | A | 1 |
| *AhLOX7_E2* | 1,713 | - | - | - | 2 |
| *AhLOX7_F1* | 1,714 | - | - | A | 3 |
| *AhLOX7_C1* | 1,716 | T | A | A | 4 |

^1^The numbers indicate nucleotide positions based on distance from 5' end of the amplicon
