# Supplementary material for: TILLING by sequencing to identify induced mutations in stress resistance genes of peanut (Arachis hypogaea)
Source: BMC Genomics. 2015 Mar 7;16(1):157. doi: 10.1186/s12864-015-1348-0 (PMC4369367; doi:10.1186/s12864-015-1348-0)
Supplement: Additional file 4: Figure S13 — Alignment of all unique sequences from AhLOX7 3’ amplification (2186/2187) demonstrates sequence variation. [file 12864_2015_1348_MOESM4_ESM.pdf]

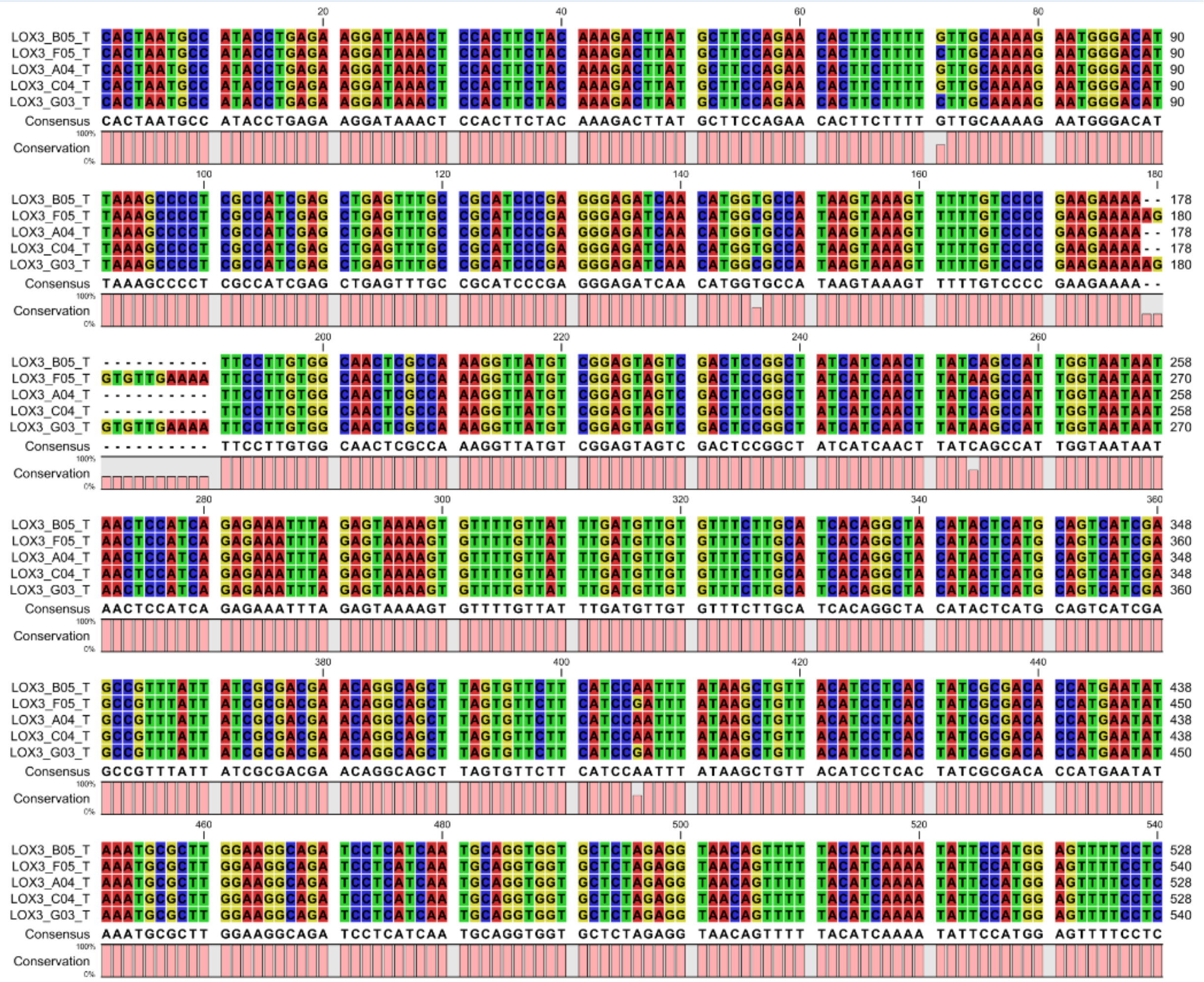
LOX3_B05_T
LOX3_F05_T
LOX3_A04_T
LOX3_C04_T
LOX3_G03_T
Consensus CACTAATGCC ATACCTGAGA AGGATAAACT CCACTTCTAC AAAGACTTAT GCTTCCAGAA CACTTCTTTT GTTGCAAAAG AATGGGACAT
Conservation
Consensus TAAAGCCCCT CGCCATCGAG CTGAGTTTGC CGCATCCCGA GGGAGATCAA CATGGTGCCA TAAGTAAAGT TTTTGTCCCC GAAGAAAA--
Consensus ---------- TTCCTTGTGG CAACTCGCCA AAGGTTATGT CGGAGTAGTC GACTCCGGCT ATCATCAACT TATCAGCCAT TGGTAATAAT
Consensus AACTCCATCA GAGAAATTTA GAGTAAAAGT GTTTTGTTAT TTGATGTTGT GTTTCTTGCA TCACAGGCTA CATACTCATG CAGTCATCGA
Consensus GCCGTTTATT ATCGCGACGA ACAGGCAGCT TAGTGTTCTT CATCCAATTT ATAAGCTGTT ACATCCTCAC TATCGCGACA CCATGAATAT
Consensus AAATGCGCTT GGAAGGCAGA TCCTCATCAA TGCAGGTGGT GCTCTAGAGG TAACAGTTTT TACATCAAAA TATTCCATGG AGTTTTCCTC

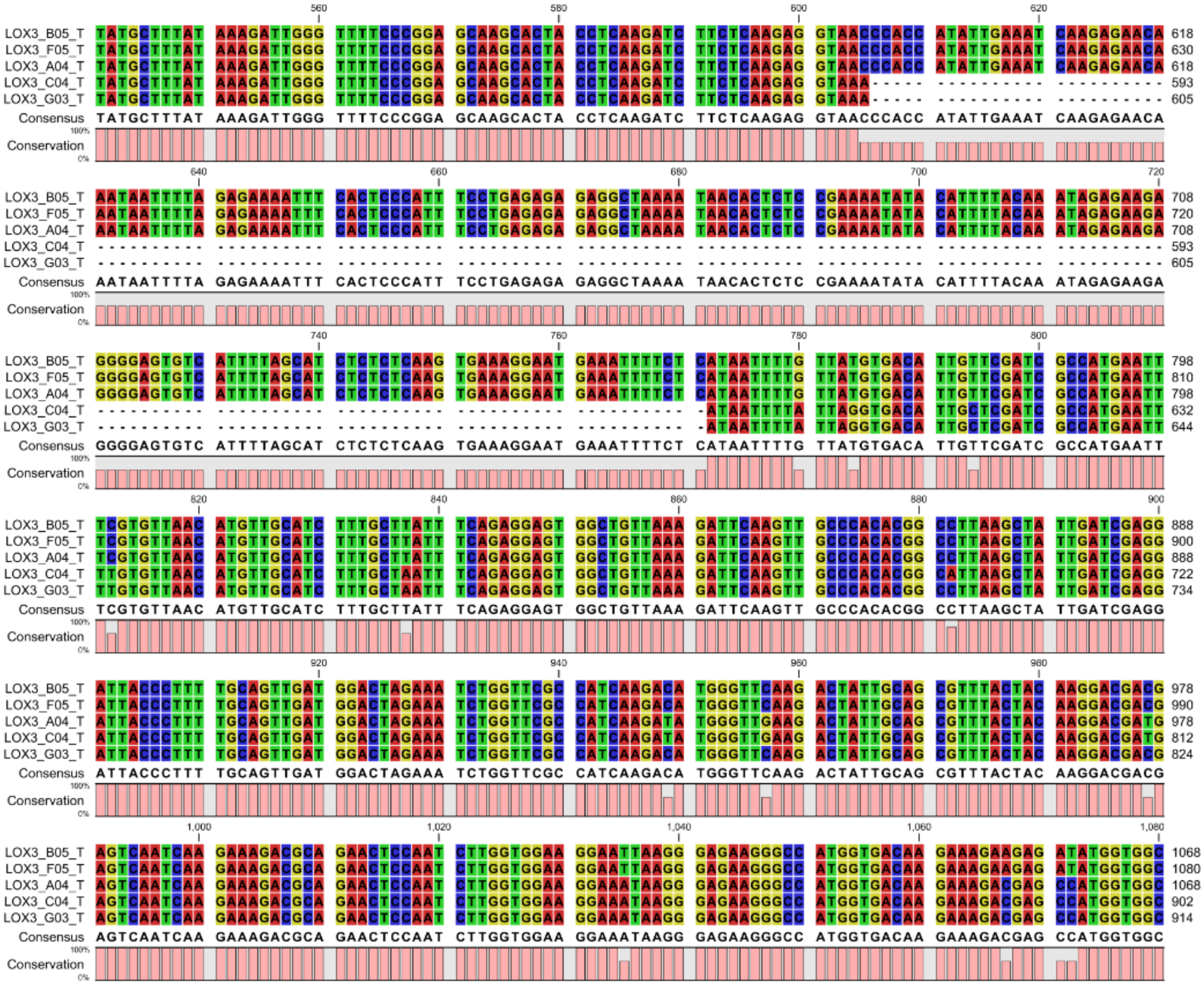
560 580 600 620
LOX3_B05_T TATGCTTTAT AAAGATTGGG TTTTCCCGGA GCAAGCACTA CCTCAAGATC TTCTCAAGAG GTAACCCACC ATATTGAAAT CAAGAGAACA 618
LOX3_F05_T TATGCTTTAT AAAGATTGGG TTTTCCCGGA GCAAGCACTA CCTCAAGATC TTCTCAAGAG GTAACCCACC ATATTGAAAT CAAGAGAACA 630
LOX3_A04_T TATGCTTTAT AAAGATTGGG TTTTCCCGGA GCAAGCACTA CCTCAAGATC TTCTCAAGAG GTAACCCACC ATATTGAAAT CAAGAGAACA 618
LOX3_C04_T TATGCTTTAT AAAGATTGGG TTTTCCCGGA GCAAGCACTA CCTCAAGATC TTCTCAAGAG GTAAA----- ---------- ---------- 593
LOX3_G03_T TATGCTTTAT AAAGATTGGG TTTTCCCGGA GCAAGCACTA CCTCAAGATC TTCTCAAGAG GTAAA----- ---------- ---------- 605
Consensus TATGCTTTAT AAAGATTGGG TTTTCCCGGA GCAAGCACTA CCTCAAGATC TTCTCAAGAG GTAACCCACC ATATTGAAAT CAAGAGAACA
100%
Conservation
0%
640 660 680 700 720
LOX3_B05_T AATAATTTTA GAGAAAATTT CACTCCCATT TCCTGAGAGA GAGGCTAAAA TAACACTCTC CGAAAATATA CATTTTACAA ATAGAGAAGA 708
LOX3_F05_T AATAATTTTA GAGAAAATTT CACTCCCATT TCCTGAGAGA GAGGCTAAAA TAACACTCTC CGAAAATATA CATTTTACAA ATAGAGAAGA 720
LOX3_A04_T AATAATTTTA GAGAAAATTT CACTCCCATT TCCTGAGAGA GAGGCTAAAA TAACACTCTC CGAAAATATA CATTTTACAA ATAGAGAAGA 708
LOX3_C04_T ---------- ---------- ---------- ---------- ---------- ---------- ---------- ---------- ---------- 593
LOX3_G03_T ---------- ---------- ---------- ---------- ---------- ---------- ---------- ---------- ---------- 605
Consensus AATAATTTTA GAGAAAATTT CACTCCCATT TCCTGAGAGA GAGGCTAAAA TAACACTCTC CGAAAATATA CATTTTACAA ATAGAGAAGA
100%
Conservation
0%
740 760 780 800
LOX3_B05_T GGGGAGTGTC ATTTTAGCAT CTCTCTCAAG TGAAAGGAAT GAAATTTTCT CATAATTTTG TTATGTGACA TTGTTCGATC GCCATGAATT 798
LOX3_F05_T GGGGAGTGTC ATTTTAGCAT CTCTCTCAAG TGAAAGGAAT GAAATTTTCT CATAATTTTG TTATGTGACA TTGTTCGATC GCCATGAATT 810
LOX3_A04_T GGGGAGTGTC ATTTTAGCAT CTCTCTCAAG TGAAAGGAAT GAAATTTTCT CATAATTTTG TTATGTGACA TTGTTCGATC GCCATGAATT 798
LOX3_C04_T ---------- ---------- ---------- ---------- ---------- -ATAATTTTA TTAGGTGACA TTGCTCGATC GCCATGAATT 632
LOX3_G03_T ---------- ---------- ---------- ---------- ---------- -ATAATTTTA TTAGGTGACA TTGCTCGATC GCCATGAATT 644
Consensus GGGGAGTGTC ATTTTAGCAT CTCTCTCAAG TGAAAGGAAT GAAATTTTCT CATAATTTTG TTATGTGACA TTGTTCGATC GCCATGAATT
100%
Conservation
0%
820 840 860 880 900
LOX3_B05_T TCGTGTTAAC ATGTTGCATC TTTGCTTATT TCAGAGGAGT GGCTGTTAAA GATTCAAGTT GCCCACACGG CCTTAAGCTA TTGATCGAGG 888
LOX3_F05_T TCGTGTTAAC ATGTTGCATC TTTGCTTATT TCAGAGGAGT GGCTGTTAAA GATTCAAGTT GCCCACACGG CCTTAAGCTA TTGATCGAGG 900
LOX3_A04_T TCGTGTTAAC ATGTTGCATC TTTGCTTATT TCAGAGGAGT GGCTGTTAAA GATTCAAGTT GCCCACACGG CCTTAAGCTA TTGATCGAGG 888
LOX3_C04_T TTGTGTTAAC ATGTTGCATC TTTGCTAATT TCAGAGGAGT GGCTGTTAAA GATTCAAGTT GCCCACACGG CATTAAGCTA TTGATCGAGG 722
LOX3_G03_T TTGTGTTAAC ATGTTGCATC TTTGCTAATT TCAGAGGAGT GGCTGTTAAA GATTCAAGTT GCCCACACGG CATTAAGCTA TTGATCGAGG 734
Consensus TCGTGTTAAC ATGTTGCATC TTTGCTTATT TCAGAGGAGT GGCTGTTAAA GATTCAAGTT GCCCACACGG CCTTAAGCTA TTGATCGAGG
100%
Conservation
0%
920 940 960 980
LOX3_B05_T ATTACCCTTT TGCAGTTGAT GGACTAGAAA TCTGGTTCGC CATCAAGACA TGGGTTCAAG ACTATTGCAG CGTTTACTAC AAGGACGACG 978
LOX3_F05_T ATTACCCTTT TGCAGTTGAT GGACTAGAAA TCTGGTTCGC CATCAAGACA TGGGTTCAAG ACTATTGCAG CGTTTACTAC AAGGACGACG 990
LOX3_A04_T ATTACCCTTT TGCAGTTGAT GGACTAGAAA TCTGGTTCGC CATCAAGATA TGGGTTGAAG ACTATTGCAG CGTTTACTAC AAGGACGATG 978
LOX3_C04_T ATTACCCTTT TGCAGTTGAT GGACTAGAAA TCTGGTTCGC CATCAAGATA TGGGTTGAAG ACTATTGCAG CGTTTACTAC AAGGACGATG 812
LOX3_G03_T ATTACCCTTT TGCAGTTGAT GGACTAGAAA TCTGGTTCGC CATCAAGACA TGGGTTCAAG ACTATTGCAG CGTTTACTAC AAGGACGACG 824
Consensus ATTACCCTTT TGCAGTTGAT GGACTAGAAA TCTGGTTCGC CATCAAGACA TGGGTTCAAG ACTATTGCAG CGTTTACTAC AAGGACGACG
100%
Conservation
0%
1,000 1,020 1,040 1,060 1,080
LOX3_B05_T AGTCAATCAA GAAAGACGCA GAACTCCAAT CTTGGTGGAA GGAATTAAGG GAGAAGGGCC ATGGTGACAA GAAAGAAGAG ATATGGTGGC 1068
LOX3_F05_T AGTCAATCAA GAAAGACGCA GAACTCCAAT CTTGGTGGAA GGAATTAAGG GAGAAGGGCC ATGGTGACAA GAAAGAAGAG ATATGGTGGC 1080
LOX3_A04_T AGTCAATCAA GAAAGACGCA GAACTCCAAT CTTGGTGGAA GGAAATAAGG GAGAAGGGCC ATGGTGACAA GAAAGACGAG CCATGGTGGC 1068
LOX3_C04_T AGTCAATCAA GAAAGACGCA GAACTCCAAT CTTGGTGGAA GGAAATAAGG GAGAAGGGCC ATGGTGACAA GAAAGACGAG CCATGGTGGC 902
LOX3_G03_T AGTCAATCAA GAAAGACGCA GAACTCCAAT CTTGGTGGAA GGAAATAAGG GAGAAGGGCC ATGGTGACAA GAAAGACGAG CCATGGTGGC 914
Consensus AGTCAATCAA GAAAGACGCA GAACTCCAAT CTTGGTGGAA GGAAATAAGG GAGAAGGGCC ATGGTGACAA GAAAGACGAG CCATGGTGGC
100%
Conservation
0%

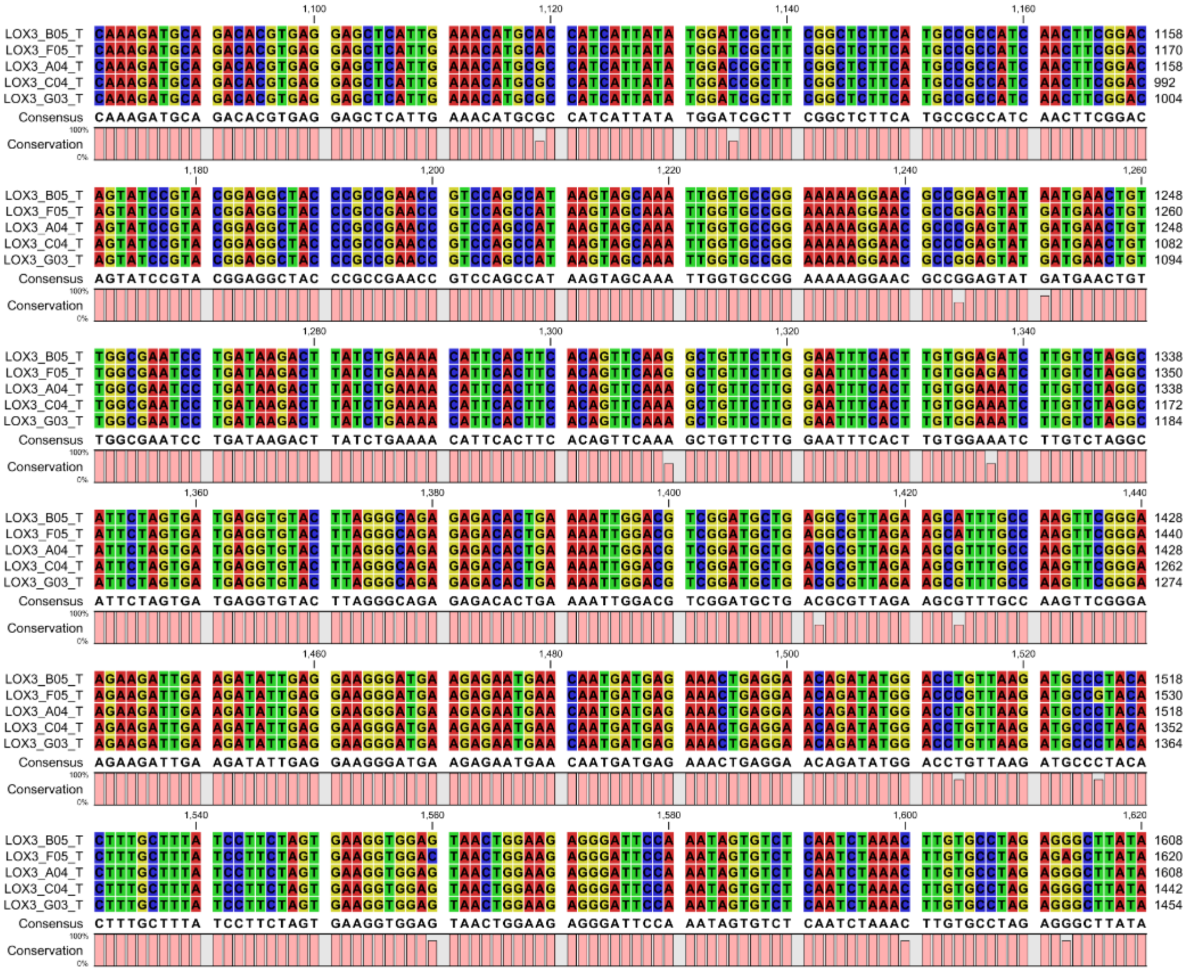

Figure S1 – Alignment of all unique sequences from *AhLOX7* 3' amplification (2186/2187) demonstrates sequence variation.
