# Supplementary material for: TILLING by sequencing to identify induced mutations in stress resistance genes of peanut (Arachis hypogaea)
Source: BMC Genomics. 2015 Mar 7;16(1):157. doi: 10.1186/s12864-015-1348-0 (PMC4369367; doi:10.1186/s12864-015-1348-0)
Supplement: Additional file 5: Figure S2. — Expression level of LOX genes in seeds and roots from RT-PCR. [file 12864_2015_1348_MOESM5_ESM.pdf]

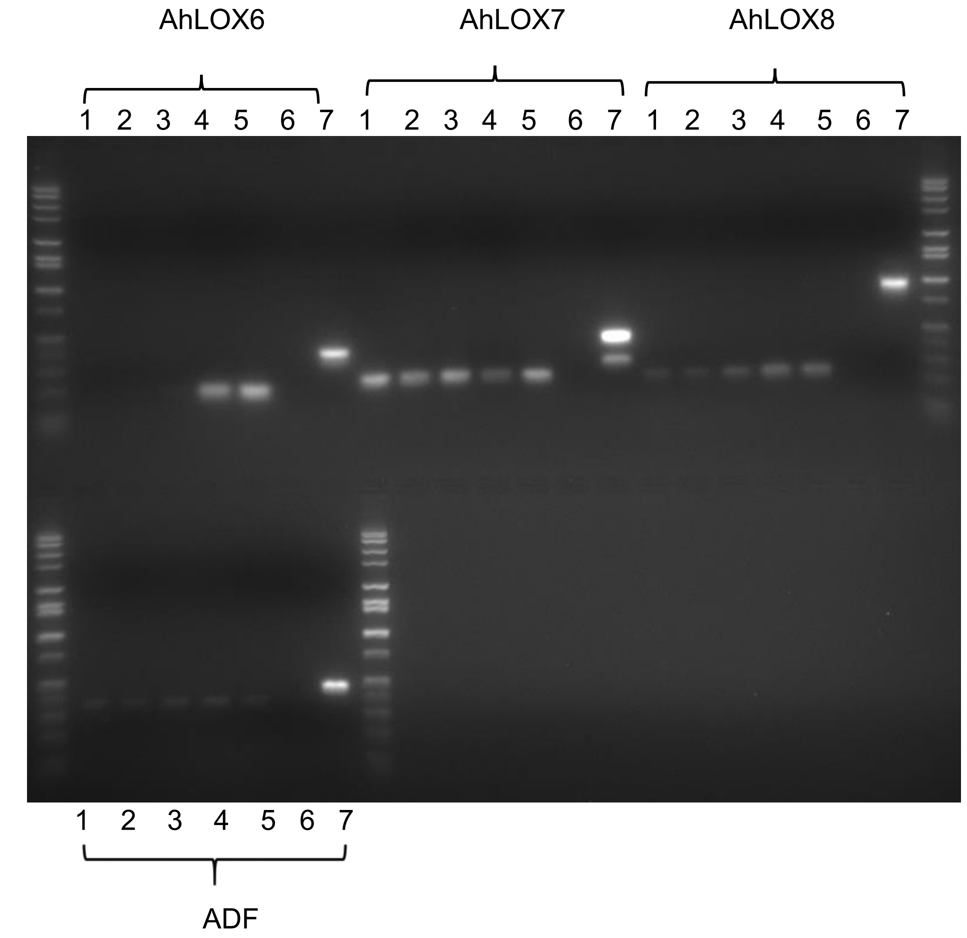

1. Tifguard seeds, yellow stage
2. Tifrunner seeds, yellow stage
3. Tifrunner seeds, orange stage
4. Tifguard isogenic line root
5. Tifrunner leaf
6. NTC
7. Genomic DNA from Tifrunner

Figure S2- Expression level of LOX genes in seeds and roots from RT-PCR. ADF- Actin depolymerizing factor gene.
