# Supplementary material for: TILLING by sequencing to identify induced mutations in stress resistance genes of peanut (Arachis hypogaea)
Source: BMC Genomics. 2015 Mar 7;16(1):157. doi: 10.1186/s12864-015-1348-0 (PMC4369367; doi:10.1186/s12864-015-1348-0)
Supplement: Additional file 6: Figure S3. — Phylogenetic analysis of all LOX genes with EST sequences. The tree was displayed in topology layout; branch labels denote lengths. [file 12864_2015_1348_MOESM6_ESM.pdf]

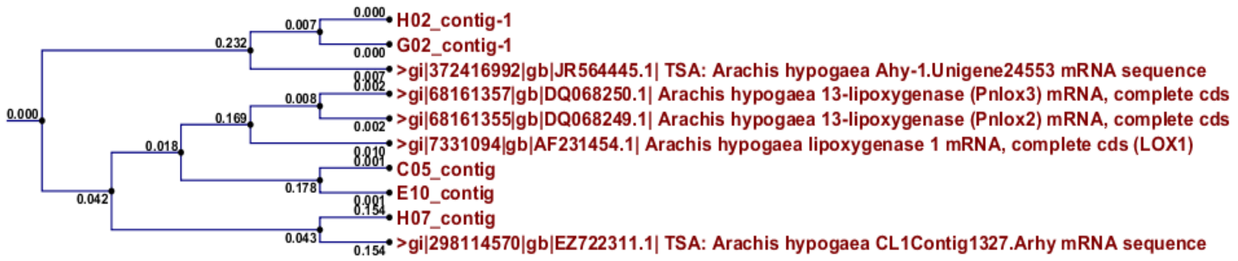

Figure S3 – Phylogenetic analysis of all LOX genes with EST sequences. The tree was displayed in topology layout; branch labels denote lengths.
