# Supplementary material for: TILLING by sequencing to identify induced mutations in stress resistance genes of peanut (Arachis hypogaea)
Source: BMC Genomics. 2015 Mar 7;16(1):157. doi: 10.1186/s12864-015-1348-0 (PMC4369367; doi:10.1186/s12864-015-1348-0)
Supplement: Additional file 7: Table S4. — Sequence variation of AhPLD1 amplicon. [file 12864_2015_1348_MOESM7_ESM.docx]

## Table S4- Sequence variation of *AhPLD1* amplicon

| Name | Length | 1126^1^ | 1778 | 1779 | 1780 | 1787 | 1793 | 1811 | 1812 | 1813 | 1839 | 1846 | 1847 | 1933 | 1955 | 1960 | 2024 | Category |
| --- | --- | --- | --- | --- | --- | --- | --- | --- | --- | --- | --- | --- | --- | --- | --- | --- | --- | --- |
| *AhPLD1_A09* | 1272 | A | A | T | G | G | C | C | G | G | A | C | C | C | A | A | T | 1 |
| *AhPLD1_E09* | 1272 | T | A | T | G | G | C | C | G | G | A | C | C | C | A | A | T | 2 |
| *AhPLD1_G08* | 1272 | A | T | G | A | A | T | G | A | A | C | T | T | A | G | G | T | 3 |
| *AhPLD1_H08* | 1271 | A | A | T | G | G | C | C | G | G | A | C | C | C | A | A | - | 4 |

^1^The numbers indicate nucleotide positions based on distance from 5' end of the amplicon
