# Supplementary material for: TILLING by sequencing to identify induced mutations in stress resistance genes of peanut (Arachis hypogaea)
Source: BMC Genomics. 2015 Mar 7;16(1):157. doi: 10.1186/s12864-015-1348-0 (PMC4369367; doi:10.1186/s12864-015-1348-0)
Supplement: Additional file 8: Table S5. — Sequence variation of AhPLD2 amplicon. [file 12864_2015_1348_MOESM8_ESM.docx]

## Table S5 - Sequence variation of *AhPLD2* amplicon

| Name | Length | 716^1^ | 739 | 2196 | 2207 | 2210 | Category |
| --- | --- | --- | --- | --- | --- | --- | --- |
| *AhPLD2_H12* | 1500 | C | G | C | C | T | 1 |
| *AhPLD2_A11* | 1500 | C | G | C | T | T | 2 |
| *AhPLD2_A12* | 1500 | C | G | C | C | A | 3 |
| *AhPLD2_B12* | 1500 | A | G | T | C | T | 4 |
| *AhPLD2_G11* | 1500 | C | T | C | C | T | 5 |

^1^The numbers indicate nucleotide positions based on distance from 5' end of the amplicon.
