# Supplementary material for: TILLING by sequencing to identify induced mutations in stress resistance genes of peanut (Arachis hypogaea)
Source: BMC Genomics. 2015 Mar 7;16(1):157. doi: 10.1186/s12864-015-1348-0 (PMC4369367; doi:10.1186/s12864-015-1348-0)
Supplement: Additional file 9: Table S6. — Mutations identified from previous study. [file 12864_2015_1348_MOESM9_ESM.docx]

## Table S6 - Mutations identified from previous study ^[39].^

| **Gene** | **Nucleotide Change** | **Predicted AA Change** | **Population** | **Plant ID** |
| --- | --- | --- | --- | --- |
|  | | | | |
| *Ara h 2.01* | G357 → A | silent | 07JKEMS1 | 65 |
| *Ara h 2.02* | G3 → A | disrupted start codon | 08GH | 2 |
| *Ara h 1.01* | C586 → T | silent | 07JKEMS1 | 99 |
| *Ara h 1.02* | C304 → T | R102 → Stop | 07JKEMS1 | 133 |
| *AhFAD2A* | C761 → T | P254 → L | 07JKEMS1 | 72 |
| *AhFAD2B* | C566 → T | silent | 07JKEMS1 | 2 |
